# Validation of the Psychometric Properties of the German Version of OBI-Care in Informal Caregivers of Stroke Survivors

**DOI:** 10.3390/jcm14176270

**Published:** 2025-09-05

**Authors:** Michael Schön, Cornelia Lischka, Hanna Köttl, Mandana Fallahpour, Susanne Guidetti, Larisa Baciu, Stefanie Lentner, Evelyn Haberl, Mona Dür

**Affiliations:** 1 Duervation GmbH, 3500 Krems, Austria; michael.schoen@duervation.com (M.S.); cornelia.lischka@duervation.com (C.L.); 2 Institute Therapeutic and Midwifery Sciences, IMC Krems University of Applied Sciences, 3500 Krems, Austria; hanna.koettl@imc.ac.at (H.K.); larisa.baciu@imc.ac.at (L.B.); stefanie.lentner@imc.ac.at (S.L.); evelyn.haberl@imc.ac.at (E.H.); 3 Department of Neurobiology, Care Sciences and Society, Division of Occupational Therapy, Karolinska Institutet, 14183 Stockholm, Sweden; mandana.fallahpour@ki.se (M.F.); susanne.guidetti@ki.se (S.G.); 4 Medical Unit Allied Health Professionals, Women’s Health and Allied Health Professionals Theme, Karolinska University Hospital, 17176 Stockholm, Sweden

**Keywords:** occupational therapy, occupational balance, occupational contingency, informal caregivers, stroke, psychometric properties, measurement instrument, ADL

## Abstract

**Background**: In occupational science and therapy, occupations are understood as meaningful activities. Satisfaction with the amount and variety of occupations is called occupational balance. The “Occupational Balance in Informal Caregivers” (OBI-Care) questionnaire assesses satisfaction with occupations across three subscales: occupational areas, characteristics, and resilience. In doing so, it also addresses occupational contingency, i.e., the ability to adapt occupations in response to unforeseen events. While previous studies have confirmed its validity in other populations, psychometric properties have not been explored in informal caregivers of stroke survivors. This study aimed to evaluate the construct validity, internal consistency, and suitability of the German OBI-Care for assessing occupational balance for this target group. **Methods:** A validation study was conducted using data collected via an online survey. Measurement properties of the three subscales were examined using Rasch Rating Scale analysis, exploring construct validity, internal consistency, and interpretability. Construct validity was assessed via dimensionality analyses, item fit, model fit, and threshold ordering. Internal consistency was evaluated using inter-item correlations, item–total correlations, person separation index, and Cronbach’s alpha. Interpretability was examined through floor and ceiling effects. **Results:** A total of 156 informal caregivers of stroke survivors participated, with 84% (*n* = 131) women and a median age of 58 (IQR: 49–66) years. All subscales showed unidimensionality with acceptable item and model fit and ordered thresholds. Internal consistency was excellent across all subscales. No floor and ceiling effects were observed. **Conclusions:** This study demonstrates good construct validity, internal consistency, and interpretability of the German OBI-Care. It is suitable for assessing occupational balance and may help identify and support occupational contingency in informal caregivers of stroke survivors.

## 1. Introduction

Globally, around twelve million people experience a stroke every year. It is known to be one of the leading causes of long-term disabilities [1]. In addition, not only does the life of the person suffering from the stroke change rapidly, but their social environment is also highly affected [2]. In many cases, care and nursing activities are performed by informal caregivers in home environments [3]. Informal caregivers are persons providing care for someone close to them, such as a family member or a friend, without receiving payment [4,5]. This support can range from assistance with personal care and mobility to everyday tasks such as grocery shopping, organizing medical appointments, managing medications, or providing emotional support [6]. Providing informal care can be associated with both positive and negative effects. While informal caregivers may experience personal rewards and a sense of purpose [7], they can also face challenges, including physical and mental health problems. Generally, the psychological impacts are more intense than the physical ones [8], especially among individuals providing high-intensity informal care [9].

Compared to informal caregivers of older adults in general, informal caregivers of stroke survivors often face more sudden and complex caregiving demands due to the abrupt onset and potential severity of stroke-related impairments [10]. These may encompass physical impairments, cognitive deficits, speech and communication difficulties, and emotional or behavioural changes. Such challenges can emerge without warning and demand immediate adaptation from informal caregivers [11]. This study offers a structured approach to understanding the impact of caregiving on occupational balance by assessing how informal caregivers of stroke survivors perceive and adapt their occupations in response to these challenges.

According to Wilcock [12], occupation is defined as meaningful activity, essential to human survival and wellbeing, enabling individuals to meet basic needs, develop skills, and exercise personal capacities. Occupational balance is a construct grounded in occupational therapy and occupational science that is vitally important for health and wellbeing and is considered a health-promoting factor [13]. Furthermore, occupational balance is associated with health, especially subjective and mental health [14], and immunological parameters [15]. Occupational balance is defined diversely [16]. However, a common definition of occupational balance is the individual’s perception of having the right amount of meaningful occupations and the right balance between them [17]. Previous research shows that the gaps experienced between what individuals want to do and what they actually do are associated with their perceived life satisfaction [18]. Therefore, we consider occupational balance as a subjective sense of satisfaction with what one does, has to do, and wants to do.

Another relevant concept is occupational contingency, which we introduce in this study as a distinct yet related construct to occupational resilience. Traditionally, contingency has been defined as caregivers’ ability to adaptively respond to the changing needs of the care recipient [19]. Extending this perspective to an occupational framework, we conceptualise occupational contingency as the capacity to resiliently maintain, flexibly modify, or develop meaningful occupations in the face of unforeseen events (e.g., the sudden illness of the caregiver or care recipient). While occupational resilience refers more broadly to the ability to maintain or regain meaningful occupations despite adversity [20], occupational contingency emphasises short-term, situational flexibility and adaptive responsiveness to sudden change. This conceptualisation aligns with recent evidence on adaptability, continuity, and the dynamic nature of occupational engagement [21,22]. In this sense, occupational contingency can be understood as a situational mechanism that supports occupational balance by enabling informal caregivers to preserve engagement in occupations under rapidly changing circumstances. Given the subjective nature of occupational balance and occupational contingency, it is vital to assess individuals’ subjective experiences and perceptions directly. The use of Self- or Patient-Reported Outcome Measures (PROMs) is essential in healthcare research and practice to capture the perspectives of individuals on health, functioning, and wellbeing [23]. These perspectives are particularly important when assessing subjective experiences that cannot be fully captured by clinicians, such as perceived limitations or participation in daily life. PROMs contribute to person-centred care and support decision-making processes by reflecting individuals’ own perceptions of their health and needs [23]. Ensuring that PROMs are reliable, valid, and responsive is essential for accurate and meaningful measurement [24]. Reliability ensures consistency, validity confirms that the intended construct is measured, and responsiveness reflects sensitivity to change [25].

Several instruments have been developed to assess occupational balance [16], including the Occupational Balance Questionnaire (OBQ) [26], the Satisfaction with Daily Occupations and Occupational Balance (SDO-OB) questionnaire [27], and the Adolescent Occupational Balance Scale (A-OBS) [28]. The OBQ measures individuals’ perceived satisfaction with the amount and variation of occupations in their daily lives, focusing on whether engagement across different activities feels balanced and meaningful [26]. The SDO-OB assesses the extent to which an individual perceives balance in their daily occupations, capturing satisfaction with time use and engagement across a range of everyday activities [27]. The A-OBS, designed for adolescents, evaluates perceptions of balance between school, leisure, rest, and social activities [28]. To date, the Occupational Balance in Informal Caregivers (OBI-Care) questionnaire is the only self-reporting measurement instrument specifically developed to assess occupational balance in informal caregivers [29]. It has demonstrated psychometric validity and reliability in different caregiver populations, including parents of preterm infants and caregivers of individuals with diverse health conditions [29,30]. Given the specific challenges faced by informal caregivers of stroke survivors, it is essential to evaluate whether the measurement instrument provides reliable and valid measurement in this context. To our knowledge, except for OBI-Care, there is no measurement instrument specifically addressing occupational contingency.

However, there is one OBI-Care study that included informal caregivers of stroke survivors only as part of heterogeneous samples [30], in which they represented a small proportion. For the use of a measurement instrument in intervention studies and to ensure its validity in both research and clinical practice with informal caregivers of stroke survivors, it is essential to establish its psychometric properties specifically in this target group. Therefore, this study aimed to evaluate the construct validity and internal consistency of the German version of OBI-Care, and to determine its suitability for capturing occupational balance in this target group, which is an essential step toward ensuring the measurement instrument’s applicability across different caregiving contexts.

## 2. Materials and Methods

### 2.1. Study Design

This study employed a psychometric validation approach [31] using an online survey and Rasch Rating Scale Model (RSM) analysis based on item response theory [32]. The current study was part of a larger research project, the Collaborative Research on Occupational Balance (CROB), funded by the Gesellschaft für Forschungsförderung Niederösterreich m.b.H. (FTI21-P-005).

### 2.2. Ethical Considerations

The study was approved by the ethics committee of Lower Austria (GS1-EK-4/852-2023). Participation was voluntary, and participants could stop at any time. Broad consent was implied through the completion and submission of the online survey. No financial incentives were provided to participants or those assisting with recruitment. Data was collected anonymously and managed in accordance with the General Data Protection Regulation (GDPR [33]). Data was used exclusively for the purposes of the research project. A corresponding study was also conducted in Sweden under similar ethical standards.

### 2.3. Data Collection

In the current study, individuals providing informal care to a stroke survivor were invited to complete an online survey. Participants had to be informal caregivers that meet the following inclusion criteria: (i) providing unpaid care to a family member, relative, or friend after a stroke; (ii) providing unpaid care for at least three months; (iii) living in Austria; and (iv) aged 18 years or older. The sample size was determined in accordance with Rasch analysis guidelines, which recommend a minimum of 10 participants per item to ensure stable item calibrations and adequate measurement precision [34].

Data for the study was collected using an online survey that included questions on sociodemographic characteristics (e.g., age, gender, education level, employment status, and living situation with the care recipient), caregiving (e.g., care duration, frequency, type of care, distance caregiving, and other individuals cared for), and characteristics of the care recipient (e.g., gender, age, and perceived level of impairment). To assess occupational balance, participants completed the German version of OBI-Care [29]. The German version of OBI-Care contains 22 items. Each item is rated on a five-point Likert scale from 1 (very satisfied) to 5 (very dissatisfied). The German version of OBI-Care’s items are grouped into three subscales that reflect different dimensions of occupational balance. Subscale 1 (Occupational Areas) measures satisfaction with the distribution of occupations across life domains, such as self-care, productivity, and leisure. Subscale 2 (Occupational Characteristics) assesses the quality and effects of occupations, including aspects like structure, meaningfulness, and perceived demands. Subscale 3 (Occupational Resilience) captures the individual’s ability to adapt their occupational engagement in response to changing circumstances. Subscales and items are described more in depth in the supplements of Dür et al. [29]. Raw item values are summed to calculate subscale scores [29]. The subscales reflect multidimensional aspects of occupational balance [29], each contributing to the overall construct. We consider subscale 3 to also reflect occupational contingency, which we defined as the capacity to flexibly sustain, modify, or develop meaningful occupations in the face of unforeseen events.

From August 2023 to April 2025, various recruitment strategies were employed. Participants were invited to take part in the study through healthcare providers (e.g., rehabilitation centres, general practitioners, occupational therapists, and neurologists), community-based organisations (e.g., Caritas and Hilfswerk), and local caregiver support groups. Digital recruitment included social media campaigns and press releases. Participants were invited to complete an online survey using the platform EFS Survey (Tivian XI GmbH, Cologne, Germany) via UNIPARK. Participants were asked to give their broad consent by clicking a confirmation button before starting the survey.

### 2.4. Data Analysis

Data from the German version of OBI-Care was exported in .xml format and processed in Microsoft Excel (Version 2506) for data validation and preparation, regardless of full survey completion. Statistical analyses were performed using RStudio (R version 4.4.1). Descriptive analyses were conducted to assess sample characteristics, including the Kolmogorov–Smirnov test [35] to explore the distribution of the data. Following the distribution, medians and interquartile ranges were calculated. Participants with incomplete OBI-Care data (one or more missing item responses) were excluded from further analysis. To examine the construct validity of each subscale of the German version of OBI-Care, data was analysed using Rasch RSM analysis [36,37,38,39,40]. RSM is a probabilistic model based on item response theory that allows for the analysis of ordinal data and provides evidence for both internal scale validity and dimensionality. The decision to apply RSM rather than the Partial Credit Model was based on the uniform response format of the German version of OBI-Care, which uses a consistent 5-point Likert scale across all items. The RSM assumes a common rating scale structure across items and provides a more efficient model when item thresholds are not expected to vary [41].

First, the dimensionality of the German version of OBI-Care was assessed through factor analysis using principal component analysis (PCA). Components with eigenvalues ≥ 1 were interpreted as independent factors. A single independent factor was considered to indicate unidimensionality; two or more factors indicated multidimensionality [42]. Following the Rasch model methodological recommendations, unidimensionality was further evaluated using person-level *t*-tests on the residuals of the first principal component. A proportion of significant *t*-tests < 5% was considered evidence for unidimensionality, while higher values indicated possible multidimensionality. Paired *t*-tests were also conducted to test for secondary dimensions. Each subscale had to be unidimensional to ensure good construct validity [43]. Additionally, the internal scale validity was assessed by examining item and person fit statistics. Item fit was assessed through item fit residuals. Acceptable item fit was defined as residuals between −2.5 and 2.5, averaging near zero with a standard deviation close to one. Non-significant infit and outfit mean square values ranging from 0.8 to 1.2 indicate an overall fit to the model [40,44]. Threshold ordering and response category functioning were examined using threshold maps and threshold probability curves. Ordered thresholds indicated appropriate functioning of the item response categories [39,40]. Internal consistency was explored using inter-item correlations, item–total correlations, Cronbach’s α, and the Person Separation Index (PSI). Inter-item correlations between 0.2 and 0.5 and Cronbach’s α between 0.70 and 0.90 indicated internal consistency. Inter-item correlations > 0.7 suggested item redundancy. Item–total correlations ≥ 0.3 and PSI ≥ 0.7 indicated the scale’s ability to distinguish between individuals [42,45]. Differential Item Functioning (DIF) was tested using the F-Test and Likelihood Ratio Test (f-statistics) to identify item bias related to age. Significance was determined at *p* < 0.05 with Bonferroni adjustment [46], and contrasts > 0.5 logits were flagged for potential bias. This approach ensured the consideration of statistical and practical aspects of DIF in the analysis, following Pallant and Tennant [40]. Interpretability was evaluated by examining floor and ceiling effects. These were considered acceptable if fewer than 15% of participants achieved the lowest or highest possible scores, indicating that the scale captured a broad range of experiences, without any data loss due to restricted response options [42,45,47].

## 3. Results

### 3.1. Participants

**Figure 1:**
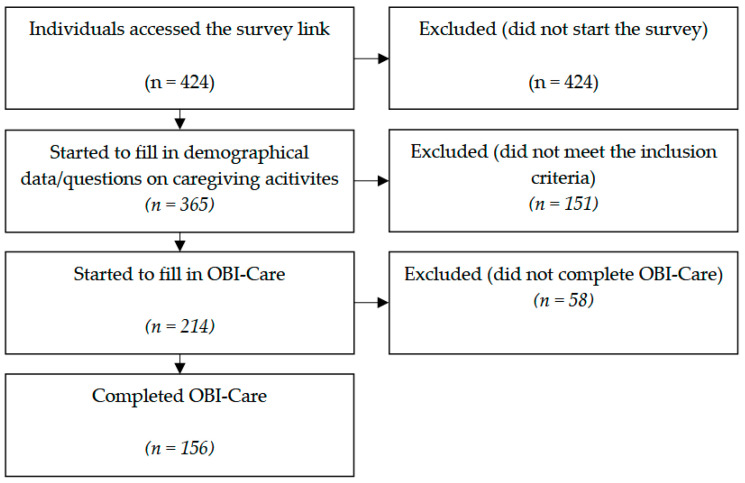
Participant flow diagram of survey respondents.

A total of 424 individuals accessed the survey link. Of these, 365 started to fill in the survey. Due to not fulfilling the inclusion criteria, 151 respondents quit before starting to fill in the German version of OBI-Care. Of the remaining 214 participants, 156 completed the OBI-Care section in the survey, resulting in a drop-out rate of 27.1%. Consequently, the responses of these 156 participants were included in the final analysis. A participant flow diagram is shown in Figure 1. The majority of the participants were female (*n* = 131, 84%), and the age of the participants ranged from 20 to 84 years, with a median age of 58 years (IQR 49–66). The sample only included informal caregivers living in Austria. All participants were 18 years or older and had provided unpaid care for a person who had experienced a stroke for at least three months.

### 3.2. Construct Validity

For the full scale, the PCA identified three components with eigenvalues ≥ 1, indicating multidimensionality. In contrast, each subscale revealed only one component with an eigenvalue ≥ 1, thereby meeting the criterion of unidimensionality. The PCA results are depicted in Table 1.

The Rasch-based person-level *t*-tests for the full scale were 19.9% significant, confirming multidimensionality. For the subscales, the proportion of significant *t*-tests was lower (subscale 1 = 16%, subscale 2 = 9.6%, subscale 3 = 12.8%), suggesting minor deviations from strict unidimensionality. However, paired *t*-tests were non-significant with *p* > 0.87.

### 3.3. Internal Consistency

Inter-item correlations indicated internal consistency for all subscales. For subscale 3, one item pair (3_a and 3_b) showed high correlations, suggesting potential redundancy. However, these correlations reflected statistical overlap rather than content similarity, since the items do not capture the same aspects. All other item pairs across subscales 1 and 2, as well as the remaining items of subscale 3, stayed within the acceptable range (<0.70). All items demonstrated adequate item–total correlations (≥0.30). The correlation coefficients are presented in Table 2.

Cronbach’s α values ranged from 0.87 to 0.88 for the subscales, indicating good internal consistency across all subscales. The PSI values ranged from 0.87 to 0.88, all exceeding the 0.70 threshold and confirming the scales’ ability to differentiate between respondents with varying levels of the construct. Item-level residuals were low across all subscales (average residuals 6.56 × 10^−5^), suggesting a good model fit. Item parameters are presented in Table 3.

A small number of category thresholds initially exhibited DIF, as indicated by z-statistics exceeding the unadjusted critical values, suggesting age-related shifts in threshold locations. However, after applying the Bonferroni correction, all DIF flags were rendered non-significant, and responses showed no evidence of DIF.

A total of 17 out of 22 items showed acceptable fit, with infit and outfit values falling within the recommended range of 0.8 to 1.2. However, five items exhibited slight misfit. Item 1_h showed minor overfit (infit = 1.39, outfit = 1.35), suggesting overdispersion. Additionally, item 2_f and item 3_f showed slight overfit (e.g., item 2_f: infit = 1.29, outfit = 1.27), while item 2_g and item 3_b fell below the lower threshold (e.g., item 2_g: infit = 0.73, outfit = 0.72), indicating potential redundancy or overly predictable response patterns. The infit and outfit values are presented in Table 4.

All items of each subscale showed ordered thresholds. All four response thresholds (τ1–τ4) increased monotonically for each item. Moreover, all response categories were used. The corresponding threshold map is presented in the Supplementary Materials.

### 3.4. Floor and Ceiling Effects

No ceiling effects were observed for the full scale or any of the subscales. Floor effects remained below the critical threshold of 15% for all subscales, with 1.28% for subscale 1, 4.49% for subscale 2, and 5.77% for subscale 3, indicating good interpretability and appropriate targeting of item difficulty for the population.

## 4. Discussion

The significance of informal caregivers’ maintenance and enhancement of meaningful occupations was emphasised previously in occupational science literature [48], which provides the theoretical base for understanding occupational balance. Building on this knowledge base, occupational therapy has an important role in supporting informal caregivers to engage in and sustain such occupations [49]. A reliable and valid measurement instrument to assess occupational balance is crucial for evaluating the effectiveness of targeted interventions.

Although occupational balance of stroke survivors has been explored in previous studies [50], to date, there has been no quantitative assessment of occupational balance in this specific caregiver population, and the German version of OBI-Care has not yet been validated for use within this group. The current study expands the research on OBI-Care by examining the psychometric properties of the German version of OBI-Care in informal caregivers of stroke survivors, thereby contributing to a broad application of the German version of OBI-Care.

The three subscales of the German version of OBI-Care portrayed unidimensionality in the PCA analysis, which fits with the structure of the questionnaire, as each subscale is designed to measure a distinct aspect of occupational balance. Complementing the PCA findings, Rasch-based person-level *t*-tests indicated that while the full scale showed clear multidimensionality, the subscales showed lower proportions. Although these values exceed the ideal 5% threshold, paired *t*-tests were non-significant, suggesting no evidence of a systematic second dimension. Taken together, these results support the interpretation of each subscale as essentially unidimensional. Importantly, this also confirms that OBI-Care should not be used to compute a single total score. Instead, interpretation and analysis should be conducted at the subscale level. These results align with a previous investigation of OBI-Care in parents of preterm infants, where similar patterns of subscale unidimensionality and overall multidimensionality were reported [29]. In contrast, a validation study in a more heterogeneous caregiver population showed multidimensionality even within subscale 1 [30]. Focusing on a specific target group, rather than a heterogeneous sample, likely contributed to the improved model fit observed in the present study [32]. Overall, the results are consistent with expectations and support the robustness of the measurement instrument in this specific population.

The Cronbach’s Alpha values for all three subscales (ranging from 0.87 to 0.88) indicate strong internal consistency, suggesting that the items within each subscale reliably measure the intended construct [51]. The distinction of subscales also aligns with previous findings on occupational balance [20,52], emphasising the importance of addressing multiple dimensions when evaluating informal caregiver wellbeing [53].

A total of 17 out of 22 items demonstrated acceptable item fit, with infit and outfit values falling within the commonly recommended range of 0.8 to 1.2 [54]. Five items showed slight deviations from these thresholds. Item 1_h, addressing satisfaction with the frequency and duration of sleep, exhibited marginal misfit, indicating overdispersion, meaning that there was greater response variability than would be expected under the Rasch model. Items 2_f and 3_f displayed slight overfit, suggesting that participant responses were more predictable than anticipated. They concerned satisfaction with the balance between cognitively demanding and less demanding tasks, as well as satisfaction with the options to find new meaningful occupations with changing life circumstances. The slight overfit observed for these items could suggest that the content was interpreted in a consistent and potentially narrow manner, reducing response variability [55]. In contrast, items 2_g and 3_b fell below the lower boundary of the acceptable range, which could indicate item redundancy or content that elicits highly uniform response patterns. Items 2_g and 3_b focus on the perceived balance between indoor and outdoor occupations and the ability to redistribute time across activities in response to life changes. The underfit observed for these items may point to conceptual overlap with other items or an overly deterministic phrasing that limited variation in responses. It is important to emphasise that the observed deviations were minor, and all items would fall within the more lenient fit range of 0.7 to 1.3 proposed in literature [56]. Therefore, we opted to retain all five items, as they were considered theoretically relevant and relevant for the participants involved in the development of OBI-Care [29] and did not show evidence of redundancy. While the results do not indicate serious psychometric concerns, they highlight areas for potential improvement. Specifically, the items in question could benefit from closer examination and potential revision to ensure sufficient conceptual clarity and avoid redundancy, thereby enhancing their contribution to the overall measurement model.

Moreover, if issues with the infit and outfit values of these items arise in potential future studies, we suggest evaluating them based on qualitative approaches (e.g., cognitive interviews and think-aloud protocols) to examine response behaviour in more depth and guide decisions regarding revision, clarification, or removal [57].

The DIF analyses showed no significant differences across age groups, indicating that the questionnaire items are fair and unbiased regardless of age [58]. This supports the applicability of the German version of OBI-Care across different age groups, enhancing its generalisability and suitability in clinical and research settings. The lack of DIF contrast suggests that differences in scores are likely due to genuine variations in occupational balance rather than age [32]. Given that the participants in the current study ranged in age from 20 to 84 years, with a median age of 58, it reflects the population of informal caregivers well [59]. However, further research involving a younger cohort could be valuable to validate the German version of OBI-Care among informal caregivers of stroke survivors within a younger age group, especially as 2% to 8% of individuals under 25, so-called young carers, regularly provide unpaid care to a family member or friend [60].

Inter-item correlations showed reliable results, except for items 3_a and 3_b (inter-item correlation = 0.75), indicating a strong relationship between the items. Both items address flexibility within occupations in case of changed circumstances, specifically, the ability to change the order of occupations and the ability to spend more time on some occupations and less time on others [29]. Hence, they show similarity but no redundancy. In contrast to previous findings that suggested redundancy between items 3_c and 3_d [29,30], these items met all psychometric criteria in the present study. Röschel et al. [30] also detected high inter-item correlations for the items 3_a and 3_b (as in this study) and 3_e and 3_f. We believe that the focus on a specific and homogeneous target group in our study contributed to the improved clarity and psychometric consistency of the results. This may explain the differences observed when compared to a study involving a mixed population of informal caregivers [30].

The low floor and ceiling effects across all subscales suggest that the questionnaire effectively captures the variability in occupational balance experiences without bias toward extreme scores [61]. This is particularly important for detecting subtle differences in occupational balance in informal caregivers who may be experiencing varying levels of burden or have different needs for care [15].

OBI-Care is the first measurement instrument that investigates occupational resilience regarding occupational balance [29]. Occupational resilience broadly refers to the ability to adapt to unforeseen changes, which is a central challenge in informal caregiving [62]. While unpredictability can disrupt daily routines and impact occupational balance [63], we conceptualise occupational contingency as a specific and vital component of occupational resilience. It describes the capacity to flexibly adjust or reorganise meaningful occupations in response to acute, unforeseen events. This distinction is particularly relevant for informal caregivers of stroke survivors, who frequently encounter unpredictable, disruptive events [64]. Occupational contingency thus reflects the situational, immediate dimension of resilience, requiring direct adaptation to maintain occupational balance. Recognising occupational contingency as a distinct aspect of occupational resilience emphasises the need to specifically assess and support caregivers’ abilities to manage unforeseen disruptions. Using robust self-reported outcome measures, or PROMs [23], is essential to capture these experiences and tailor interventions that sustain caregivers’ wellbeing and occupational balance.

The results show that the German version of OBI-Care is a valid and reliable measurement instrument that can be used in practical settings to assess the occupational balance in informal caregivers of stroke survivors. Validation in this population is essential, as stroke caregiving is often sudden, intensive, and long-term, creating distinct challenges compared to other caregiving contexts. Having a validated measurement instrument ensures that clinicians can confidently use the German version of OBI-Care to identify restricted occupational balance specific to this group. This enables health professionals to design tailored support strategies, which may help reduce caregiver burden, improve caregivers’ health outcomes, and ultimately sustain the quality of informal care. Beyond occupational balance, the measurement instrument also provides a basis for exploring occupational contingency, which highlights informal caregivers’ situational adaptability in sustaining meaningful occupations. Identifying imbalances in informal caregivers’ occupations can enable clinicians to tailor support measures to individual needs, which may help reduce caregiver burden, improve health outcomes, and strengthen the sustainability of informal care arrangements. Previous literature suggests that a good occupational balance can have a positive impact on the health of the informal caregiver as well as the person being cared for [65,66]. Thus, it is recommended that healthcare practitioners support informal caregivers of stroke survivors in maintaining or enhancing their occupational balance. The use of the German version of OBI-Care in a practical setting should therefore be promoted in the future. Furthermore, after the validation of the German version of OBI-Care in this specific group, we suggest including it in future research addressing informal caregivers of stroke survivors.

### Limitations and Future Directions

While the study confirms the reliability and validity of the German version of OBI-Care, some limitations should be acknowledged. In alignment with the high proportion of women in informal caregiving in general [67], our sample predominantly consisted of female participants, which may limit the generalisability of the findings to male caregivers. While this gender imbalance limited our ability to test for DIF by gender, it accurately reflects real-world caregiving demographics [68]. Nevertheless, confirming that the German version of OBI-Care functions equivalently across genders remains important. Future research should therefore include a more balanced sample to assess whether the psychometric properties hold across different gender distributions.

Second, as the questionnaire was administered online, only individuals with internet access and digital devices were able to participate, unintentionally excluding informal caregivers with lower levels of digital affinity, possibly affecting the age distribution as well. The overall response rate in our study was 36.8%, which is slightly lower than the average reported in online survey research (~44%) [69]. Although our response rate is comparable to many online health surveys, limited participation may still reduce generalisability. While lower response rates can pose a risk of bias and affect data quality [70], our findings remain consistent with expected ranges in similar contexts, as informal caregivers are a difficult-to-access group for research [71]. It is possible that informal caregivers with heavier workloads were less likely to complete the survey; however, evidence suggests that highly involved informal caregivers may actually be more likely to participate [72], so the direction of this potential bias remains uncertain. Given this uncertainty, it remains important to explore participation patterns in more detail and consider strategies that may improve inclusivity in future studies. Future studies could examine different groups of informal caregivers, such as people living in rural areas or groups that show less digital health literacy, or offer a pen-and-paper version of the questionnaire to enhance accessibility and inclusivity.

Third, according to Linacre [73], the sample size of 156 participants was small in relation to the 22 items of the German version of OBI-Care. However, there is evidence that sample sizes of 100 participants already allow reliable and valid statistics [74]. Furthermore, due to the small number of participants, chi-square tests were not conducted, and instead infit and outfit values were included for further interpretation. Future studies should address items falling outside the acceptable infit/outfit range using qualitative methods such as cognitive interviews or think-aloud protocols. These approaches would clarify how participants interpret and distinguish items, particularly those with high inter-item correlations. In addition, future research should examine whether demographic and health-related characteristics, such as age, chronic illness, or perceived health status, may have influenced item performance in this study, particularly for items that showed underfit compared to earlier validation results.

Finally, the present study validated only the German version of OBI-Care. As a next step, validating versions in different languages would significantly broaden the measurement instrument’s applicability, enabling its use in larger populations. A similar study is currently being conducted in Sweden as part of the same research project, further supporting the international relevance and interest in this measurement instrument.

## 5. Conclusions

The present study demonstrates that the German version of OBI-Care is a reliable and valid measurement instrument for assessing the occupational balance in informal caregivers of stroke survivors. The strong psychometric properties, including high internal consistency, good model fit, and the absence of DIF, support its use in this target group. The multidimensional structure confirms that the measurement instrument should be evaluated at the subscale level rather than as a total score. By capturing occupational areas, characteristics, and resilience, the instrument reflects different facets of occupational balance that are closely linked to caregivers’ capacity to adapt to changing circumstances. This connection underlines the relevance of occupational contingency as an additional factor in maintaining a balance of meaningful occupations. The German version of OBI-Care therefore provides a sound basis for both research and clinical applications in caregivers of stroke survivors.

## Figures and Tables

**Table 1:** Dimensionality of the German version of OBI-Care extracted via principal component analysis (PCA).

Subscale 1				Subscale 2				Subscale 3			
Item	Eigenvalue			Item	Eigenvalue			Item	Eigenvalue		
	Total	% of VA	CUM %		Total	% of VA	CUM %		Total	% of VA	CUM %
**1_a**	4.55	50.58	50.58	**2_a**	3.93	56.20	56.20	**3_a**	3.74	62.25	62.25
**1_b**	0.95	10.52	61.10	**2_b**	0.83	11.88	68.08	**3_b**	0.81	13.42	75.67
**1_c**	0.71	7.86	68.96	**2_c**	0.59	8.37	76.45	**3_c**	0.56	9.28	84.95
**1_d**	0.63	7.00	75.96	**2_d**	0.56	7.94	84.39	**3_d**	0.37	6.12	91.06
**1_e**	0.53	5.93	81.89	**2_e**	0.43	6.15	90.54	**3_e**	0.33	5.47	96.53
**1_f**	0.46	5.14	87.03	**2_f**	0.36	5.14	95.67	**3_f**	0.21	3.47	100.00
**1_g**	0.44	4.93	91.96	**2_g**	0.30	4.33	100.00				
**1_h**	0.38	4.27	96.23								
**1_i**	0.34	3.77	100.00								

Abbreviations: CUM = cumulative; VA = variance; OBI-Care = Occupational Balance in Informal Caregivers.

**Table 2:** Inter-item correlation and total–item correlation of each subscale of the German version of OBI-Care.

**Subscale 1**	**Inter-Item Correlation**									**Total–Item** **Correlation**	
	**1_a**	**1_b**	**1_c**	**1_d**	**1_e**	**1_f**	**1_g**	**1_h**	**1_i**		
**1_a**	1.00	0.51	0.47	0.40	0.43	0.46	0.44	0.27	0.34	**1_a**	**0.65**
**1_b**	0.51	1.00	0.60	0.40	0.39	0.45	0.38	0.34	0.41	**1_b**	0.69
**1_c**	0.47	0.60	1.00	0.43	0.32	0.46	0.37	0.36	0.46	**1_c**	0.69
**1_d**	0.40	0.40	0.43	1.00	0.50	0.54	0.44	0.45	0.46	**1_d**	0.73
**1_e**	0.43	0.39	0.32	0.50	1.00	0.52	0.48	0.45	0.45	**1_e**	0.72
**1_f**	0.46	0.45	0.46	0.54	0.52	1.00	0.61	0.44	0.50	**1_f**	0.78
**1_g**	0.44	0.38	0.37	0.44	0.48	0.61	1.00	0.40	0.55	**1_g**	0.73
**1_h**	0.27	0.34	0.36	0.45	0.45	0.44	0.40	1.00	0.48	**1_h**	0.67
**1_i**	0.34	0.41	0.46	0.46	0.45	0.50	0.55	0.48	1.00	**1_i**	0.73
**Subscale 2**	**Inter-Item Correlation**									**Total–Item** **Correlation**	
	**2_a**	**2_b**	**2_c**	**2_d**	**2_e**	**2_f**	**2_g**				
**2_a**	1.00	0.60	0.41	0.46	0.44	0.37	0.46			**2_a**	**0.71**
**2_b**	0.60	1.00	0.50	0.49	0.39	0.40	0.45			**2_b**	0.74
**2_c**	0.41	0.50	1.00	0.48	0.46	0.40	0.51			**2_c**	0.71
**2_d**	0.46	0.49	0.48	1.00	0.59	0.48	0.60			**2_d**	0.79
**2_e**	0.44	0.39	0.46	0.59	1.00	0.48	0.65			**2_e**	0.76
**2_f**	0.37	0.40	0.40	0.48	0.48	1.00	0.62			**2_f**	0.72
**2_g**	0.46	0.45	0.51	0.60	0.65	0.62	1.00			**2_g**	0.82
**Subscale 3**	**Inter-Item Correlation**									**Total–Item** **Correlation**	
	**3_a**	**3_b**	**3_c**	**3_d**	**3_e**	**3_f**					
**3_a**	1.00	0.75	0.50	0.43	0.55	0.49				**3_a**	0.78
**3_b**	0.75	1.00	0.53	0.41	0.68	0.54				**3_b**	0.82
**3_c**	0.50	0.53	1.00	0.67	0.51	0.52				**3_c**	0.78
**3_d**	0.43	0.41	0.67	1.00	0.50	0.52				**3_d**	0.74
**3_e**	0.55	0.68	0.51	0.50	1.00	0.63				**3_e**	0.83
**3_f**	0.49	0.54	0.52	0.52	0.63	1.00				**3_f**	0.79

Abbreviations: OBI-Care = Occupational Balance in Informal Caregivers.

**Table 3:** Item parameters, model fit indices (average residuals), and age-based differential item functioning (F-statistics) for each subscale of the German version of OBI-Care.

**Subscale 1**	**Location**	**SE**	**Av. residual**
**1_a**	0.66	0.10	1.33 × 10^−4^
**1_b**	0.72	0.10	1.35 × 10^−4^
**1_c**	0.24	0.10	1.19 × 10^−4^
**1_d**	−0.26	0.10	2.09 × 10^−5^
**1_e**	−0.10	0.10	2.17 × 10^−5^
**1_f**	−0.72	0.10	4.12 × 10^−5^
**1_g**	−0.37	0.10	2.03 × 10^−5^
**1_h**	0.03	0.10	1.08 × 10^−4^
**1_i**	0.04	0.10	1.08 × 10^−4^
**Subscale 2**	**Location**	**SE**	**Av. residual**
**2_a**	−0.15	0.11	1.29 × 10^−4^
**2_b**	−0.36	0.11	1.41 × 10^−4^
**2_c**	−0.08	0.11	5.91 × 10^−6^
**2_d**	−0.22	0.11	1.33 × 10^−4^
**2_e**	0.45	0.11	2.58 × 10^−6^
**2_f**	0.70	0.11	1.25 × 10^−6^
**2_g**	0.59	0.11	1.83 × 10^−6^
**Subscale 3**	**Location**	**SE**	**Av. residual**
**2_a**	−0.05	0.11	1.60 × 10^−5^
**2_b**	−0.08	0.11	2.23 × 10^−5^
**2_c**	0.24	0.11	9.37 × 10^−5^
**2_d**	0.01	0.11	8.79 × 10^−5^
**2_e**	0.01	0.11	8.79 × 10^−5^
**2_f**	−0.41	0.11	1.47 × 10^−5^

Abbreviations: OBI-Care = Occupational Balance in Informal Caregivers.

**Table 4:** Infit and outfit values of each subscale of the German version of OBI-Care.

Subscale 1	Infit	Outfit	Subscale 2	Infit	Outfit	Subscale 3	Infit	Outfit
1_a	0.85	0.85	2_a	1.06	1.08	3_a	0.97	0.97
1_b	0.93	0.93	2_b	1.16	1.14	3_b	**0.79**	**0.78**
1_c	0.90	0.91	2_c	0.90	0.93	3_c	0.88	0.90
1_d	1.06	1.05	2_d	0.95	0.94	3_d	1.10	1.08
1_e	1.17	1.16	2_e	0.94	0.94	3_e	1.06	1.04
1_f	0.87	0.85	2_f	**1.29**	**1.27**	3_f	**1.22**	1.20
1_g	0.90	0.90	2_g	**0.73**	**0.72**			
1_h	**1.39**	**1.35**						
1_i	0.99	0.98						

Abbreviations: OBI-Care = Occupational Balance in Informal Caregivers.

## Data Availability

The original contributions presented in the study are included in the article and the Supplementary Materials; further enquiries can be directed to the corresponding authors.

## Supplementary Materials

The following supporting information can be downloaded at https://www.mdpi.com/article/10.3390/jcm14176270/s1.

## Abbreviations

The following abbreviations are used in this manuscript:

A-OBS	Adolescent Occupational Balance Scale
CROB	Collaborative Research on Occupational Balance
DIF	Differential Item Functioning
GDPR	General Data Protection Regulation
IQR	Interquartile Range
OBI-Care	Occupational Balance in Informal Caregivers
OBQ	Occupational Balance Questionnaire
PCA	Principal Component Analysis
PROM	Patient Reported Outcome Measure
PSI	Person Separation Index
RSM	Rating Scale Model
SDO-OB	Satisfaction with Daily Occupations and Occupational Balance questionnaire
